# All-Cause Mortality Risk Associated With Solid Fuel Use Among Chinese Elderly People: A National Retrospective Longitudinal Study

**DOI:** 10.3389/fpubh.2021.741637

**Published:** 2021-10-14

**Authors:** Shisi Shen, Min Luo, Xuchen Meng, Ying Deng, Shuwen Cheng

**Affiliations:** ^1^The First School of Clinical Medicine, Chongqing Medical University, Chongqing, China; ^2^Department of Urology, West China Hospital, Sichuan University, Chengdu, China; ^3^Department of Chronic and Non-communicable Disease Control and Prevention, Sichuan Center for Disease Control and Prevention, Chengdu, China

**Keywords:** mortality, solid fuel, CLHLS, elderly, PM_2.5_

## Abstract

**Background:** The adverse health effects of air pollutants are widely reported, and the elderly are susceptible to toxic environments. This study aimed to evaluate the association between use of solid fuels for cooking and mortality among the elderly.

**Methods:** A total of 5,732 and 3,869 participants from the Chinese Longitudinal Healthy Longevity Survey were enrolled in two (2014 and 2018) and three surveys (2011, 2014, and 2018) of survey. Cooking fuel was divided into clean and solid fuel. Cox proportional hazards models were used to estimate the mortality hazard ratio (HR). Subgroup analyses were performed to assess the potential interaction effect.

**Results:** Among the participants in the 2011–2018 survey, 53% reported using solid fuel. Such group was associated with a 9% increase in mortality risk relative to clean fuel users (HR = 1.09, 95% CI = 1.01–1.18). Among participants in the 2014–2018 survey, 339 reported a switch from solid to clean fuels and they were not at increased mortality risk relative to the 488 people that reported a stable use of clean fuels (HR = 1.14, 95% CI = 0.99–1.31) although the estimated HR was similar to the one for stable solid fuel users (HR = 1.19, 95%CI = 1.04–1.36 *n* = 509). Interaction and stratified analyses showed that solid fuel use had an impact on mortality in participants who were non-current smokers, had low dietary diversity scores, and were living in areas with high PM_2.5_ concentrations (>50 μg/m^3^) and city population below 8 million (*P* for interaction < 0.05). The association was robust in the three sensitivity analyses.

**Conclusion:** The finding showed a clear association between solid fuel use and mortality among older Chinese, and an even stronger association between risk of mortality and solid fuel use among individuals exposed to high levels of PM_2.5_.

## Introduction

Long-term exposure to household air pollution (HAP) is a public health problem worldwide and is among the top 10 hazard factors for disease (1, 2). Solid fuel is the largest source of HAP worldwide, where nearly a third of the population in low- and middle-income countries (LMICs) rely on it for primary domestic cooking, heating, or lighting energy. In China, 450 million people are estimated to use solid fuel (3, 4). Incomplete combustion of solid fuel releases large amounts of poisonous by-products, such as carbon monoxide, polycyclic aromatic hydrocarbons (PAHs), nitrogen dioxide, and particulate matter (5), which are linked to apoplexy, ischemic heart disease, chronic obstructive pulmonary disease (COPD), pneumonia, and lung cancer (3, 4). Notably, exposure to air pollution from solid fuel burning may directly or indirectly contribute to over 4 million deaths and 110 million disabilities annually (6).

The association of solid fuel use and common diseases has been proved in many studies on the elderly (7–9). Owing to physical limitations and social factors, the elderly are generally most active in and around the house, and they willingly perform housework and spend more time on it as they age (10). A cohort study indicated that the use of biomass fuel is associated with higher hazard ratios (HRs) of hypertension among the elderly (aged ≥65 years) (7). A prospective cohort showed that long-term exposure to solid fuel and smoking can each independently increase the risk of chronic liver disease (CLD) among 501,104 participants with an average age of 52.0 years (8).

The association between solid fuel use and mortality has previously been indicated by previous studies (6, 8, 11, 12). The China Kadoorie Biobank (CKB) study reported that persistent solid fuel users had significantly higher HR of all-cause mortality and cause-mortality (cardiovascular and respiratory mortality) compared with persistent clean fuel users (12). A cohort study showed that the interaction effect of solid fuel use can modify the HR of smoking to CLD mortality (8). Although the causality of cause-mortality attributed to solid fuel use was assessed by the aforementioned studies, the validity of their evidence is limited because of outdated datasets, the transformation of developed urban-rural medical systems and the improvement of life quality. For example, the researchers involved in the CKB study visited participants from 2004 to 2008. Additionally, they did not obtain data on the association between solid fuel use and all-cause mortality in a national cohort of elderly Chinese.

The development of urbanization has greatly increased the risk of people being exposed to indoor and outdoor pollutants. A study that followed up its participants for a period of 14 years found strong association between PM_2.5_ exposure and premature mortality (13). A longitudinal study indicated that day-to-day changes in ambient PM_2.5_ concentrations are linked to high risk of all-cause mortality. Moreover, adults aged ≥85 years were the most vulnerable group to increased PM_2.5_ concentrations (14). This study highlighted the link between outdoor air pollution and mortality among the elderly. However, it failed to establish the causality between HAP and mortality. In addition, the contribution of residents to outdoor air pollution (e.g., smoking and solid fuel use) is critical. A study conducted in China in 2014 showed that residential fuel use contributes to 68% of PM_2.5_ exposure and 67% of premature deaths. Moreover, indoor pollution due to solid fuel combustion may modify and strengthen the association between traffic-related air pollution (TRAP) and cognitive impairment (15–17). Hence, the interaction between indoor and outdoor environmental factors must also be investigated. Furthermore, the effects of switching fuel used for cooking on mortality among the elderly should be examined (18).

The Chinese Longitudinal Healthy Longevity Survey (CLHLS) is an open, prospective, and national cohort of community-dwelling Chinese aged 65 years. In this study, we investigated whether the type of fuel used for cooking is associated with subsequent 8-year mortality and whether switching the fuel used for cooking for 4 years is associated with changes in HR with successive 5 years of follow-up.

## Methods

### Study Subjects

The CLHLS is a dynamic longitudinal study that began in 1998 with follow-up investigation every 2–4 years, covering 22 provinces and 85% of the elderly in China. The surveyed provinces include Liaoning, Jilin, Heilongjiang, Hebei, Beijing, Tianjing, Shanxi, Shaanxi, Shanghai, Jiangsu, Zhejiang, Anhui, Fujian, Jiangxi, Shangdong, Henan, Hubei, Hunan, Guangdong, Guangxi, Sichuan, Henan and Chongqing (19, 20). In 1998 and 2000, the respondents were over 80 years old, and since 2002, the respondents were over 65 years old. The questionnaire was divided into two types: the “survivors” and “deceased” questionnaires. All participants were interviewed on determinants of healthy longevity among elderly Chinese: socioeconomic characteristics, lifestyle, social interaction, residential environment, cognitive function, and physical and mental condition of healthy longevity among the Chinese elderly population. In each wave of survey, individuals who were still alive were interviewed again. If a participant was already dead, the “deceased” questionnaires were used, which assessed the cause of death, health condition, and health service utilization in the wave of survey. The present study included data from 2011, 2014, and 2018 survey waves of the CLHLS because the type of cooking fuel used was not incorporated in the previous questionnaires. In the 2011–2018 cohort, the first section contains the information of 9,679 respondents in 2011 after excluding 86 participants younger than 65 years. The second section contains the information from 6,009 respondents in 2014. The third section contains information on 2,879 people who died before the 2014 survey. The fourth section contains information from 2,834 respondents in 2018. The fifth section contains information on 1,833 people who died before the survey in 2018 (19) (Supplementary Figure 1). In the first wave (2011), information on cooking fuel was obtained (“Which fuel is normally used for cooking in your home?”). Follow-up surveys were conducted in 2014 and 2018.

**Figure 1:**
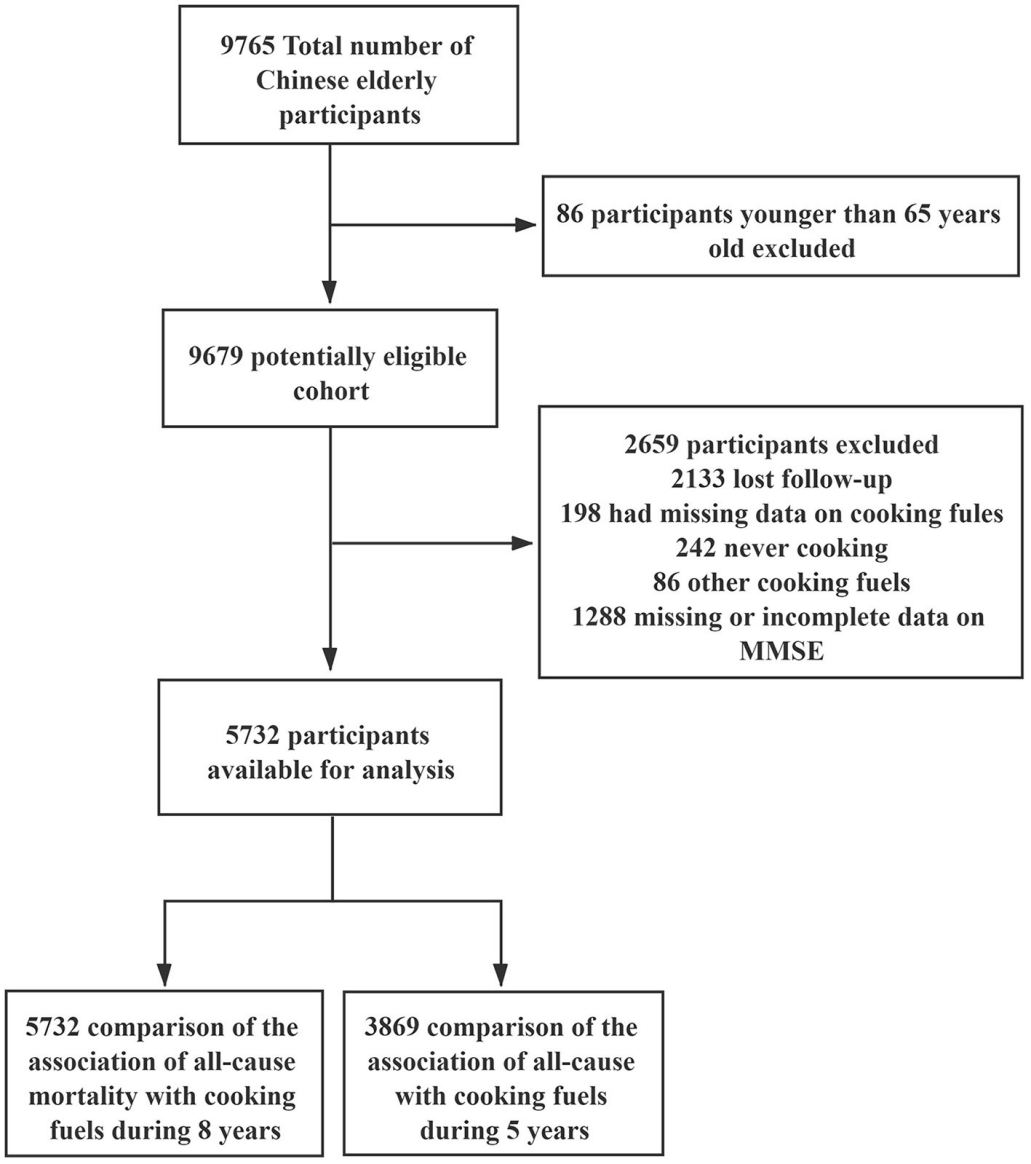
Flowchart of the inclusion of participants.

In the first wave (2011), a total of 9765 individuals were recruited. Participants were excluded if they were younger than 65 years (*n* = 86), lost during follow-up in 2014 or 2018 (*n* = 2,133), had missing data on cooking fuel (*n* = 198), never cooked (*n* = 242), used other cooking fuel (*n* = 86), or had missing or incomplete information on Mini-Mental State Examination (MMSE) (incomplete MMSE will underestimate participants' scores and increase the ratio of classification error [normal or impaired]) (*n* = 1,287). A total of 5,732 participants who were still alive were included in the analysis. The effects of switching cooking fuel on mortality were also determined. Participants who reportedly died in 2014 (*n* = 1,863) were further excluded. Finally, 3869 participants were followed up in the third wave (2018) (Figure 1).

This study was approved by the Research Ethics Committee of Peking University (IRB00001052-13074), and written consent was obtained from all participants or their legal representatives.

### Energy Source for Cooking

In all three surveys, HAP was determined by asking the participants what solid fuel they used for cooking (“Which fuel is normally used for cooking in your home?”). The energy sources for cooking were divided into two types: clean fuel and solid fuel. Clean fuel refers to liquefied gas, natural gas, and electricity, whereas solid fuel pertains to coal, charcoal, firewood, wood, and animal dung.

### Death Assessment

During the succeeding surveys in all cohorts, ascertainable date of death was obtained via interviews with family members or local doctors. Cause-specific mortality was not included in the present study because the cause and date of death were not recorded and/or unclear in a previous study (21). All interviewees were followed up from baseline until death ascertainment, lost to follow-up, or July 2019.

### Exposure

The estimates of ground-level fine particulate matter (PM_2.5_) were obtained from the Atmospheric Composition Analysis Group (22). PM_2.5_ concentrations were estimated by combining the aerosol optical depth inversion from the National Aeronautics and Space Administration's Moderate Resolution Imaging Spectroradiometer, Multi-angle Imaging SpectroRadiometer, and Sea-viewing Wide Field-of-View Sensor instruments with the GEOS-Chem chemical transport model. Geographically weighted regression was used to correct the observed value of the regional ground mass and component mass. Geophysical statistical estimates of PM_2.5_ were generated using PM_2.5_ released by the Chinese Environmental Protection Agency between May 2014 and December 2018 (Chinese EPA. https://air.cnemc.cn:18007/)^1^. It extends these values back to 2000 using the interannual changes between the GM observed and non-GM observed time periods on the basis of the V4.GL.03 geophysical satellite-derived values of (23). Population-weighted PM_2.5_ concentration is calculated according to the weight of the exposed population in the city where it is located. The weight is the proportion of the exposed population in the city to the exposed population in the province. This algorithm can reflect the impact of air pollution on residents' health more reasonably. In the 2011 Household Survey, residential locations were obtained from the basic information in the questionnaire, and the provincial information was encoded and then matched one by one using the PM_2.5_ dataset.

### Covariate Assessment

Several potential confounders were adjusted in the final analyses. Socio-demographic information, such as age, sex, residential type (rural/city), education level (illiterate/primary school/high school or higher), current marital status (never married/married), and annual family income (Yuan) (<30,000/30,000–50,000/>50,000), was collected. Lifestyle factors included smoking status (never/previously/currently), drinking status (never/previously/currently), dietary diversity scores (low/medium/high), and social activity (low/medium/high). Health status included body mass index (BMI) (underweight/normal/overweight or obese)(<18.4/18.5–24.9 />25 kg/m^2^) (24), presence of chronic diseases (e.g., hypertension, diabetes, heart disease, and stroke; yes/no), activities of daily living (ADL) (complete independence/complete dependence), instrumental activities of daily living (IADL) (complete independence/complete dependence), and cognitive function (as measured by the MMSE score) (normal/cognitive impairment). Environmental factors included population-weighted PM_2.5_ concentration (<50/≥50 μg/m^3^) and city population (>8/ ≤8 million). The detailed definition and calculation of the scores of these variables were described in other studies (15, 21, 25, 26). Detailed definitions of covariates are described in the Supplementary File (Appendix methods).

### Statistical Analysis

Continuous variables were described using mean ± standard deviation. All categorical variables were presented using number and proportion (%). Differences in categorical variables were treated by Chi-square test, and continuous variables were tested by Student's *t*-test. Two Cox proportional hazards models were used to determine the association between mortality and the type of fuel used for cooking. Clean fuel was set as the reference group. The proportional hazards assumption was performed before analysis. The first Cox regression model with baseline cooking fuel as the underlying time scale was constructed to explore the effects of long-term exposure to solid fuel on mortality for a period of 8 years. An unadjusted model was fitted. The model was adjusted for sex, age, smoking and drinking status, dietary diversity scores, BMI, marital status, education level, and social activity. The model was further adjusted for income, PM_2.5_ concentration, and city population. The final model was adjusted for ADL, IADL, MMSE, and presence of chronic diseases. The second Cox regression model was constructed to examine the association between switching cooking fuel and mortality (stable use and switching to clean fuel). The model was fitted in the same manner as described above. In addition, crude all-cause mortality (HR) was calculated across various types of fuel for cooking. The HR of covariates at the survey time t is defined as below:


$$\begin{array}{ll} h(t,X) & =h_{0}\exp(\beta_{1}age+\beta_{2}sex+\beta_{3}residence+\beta_{4}education\\ +\;\beta_{5}marital\;status+\beta_{6}income+\beta_{7}smoking\\ +\;\beta_{8}drinking+\beta_{9}dietary\;diversity\;scores+\beta_{10}BMI\\ +\;\beta_{11}physical\;activity\;scores+\beta_{12}PM_{2.5}+\beta_{13}IADL\\ \;+\beta_{14}ADL+\beta_{15}city\;population+\beta_{16}MMSE) \end{array}$$

The ‘*h(t,X*)’ is HR of mortality. The ‘*t*’ is the survival time of survivors since 2011. The ‘*X*’ is the covariates.

In the subgroup analyses, multiplicative interactions between dichotomized cooking fuel and covariates were tested by adding a cross-product term in the full multivariable Cox regression model with 8 years of follow-up period. The cross-product terms included age, sex, residency, education level, marital status, income, smoking behavior (Non–current/current), drinking behavior, dietary diversity scores, BMI, physical activity scores, PM_2.5_ concentration, IADL, ADL, city population, and MMSE. In light of significant cross-product terms and the objectives of this study, a three-way interaction analysis was further conducted to obtain quantitative insights into the association among cooking fuel, smoking, and PM_2.5_ concentration with mortality.

In sensitivity analyses, the increased mortality risk for solid fuel vs. clean fuel was still observed when individuals lost to follow-up were censored at the last wave (HR = 1.17, 95% CI = 1.08, 1.27) and when individuals who died prior to the second wave (2014) were excluded (HR = 1.28, 95% CI = 1.13, 1.45). Finally, most covariates were disproportionally and randomly missing (3–8%). Multiple imputation based on five replications and Monte Carlo simulations were used to account for missing data.

All analyses were performed using Stata 16.1 (Stata Corporation, College Station). Statistical significance was considered when *P* < 0.05 (two-sided).

## Results

### Characteristics of Study Subjects

The general information of subjects is presented in Table 1. A total of 5,732 participants were recruited during the baseline. Among them, 53.6% were females. The average age was 84.8 years. Most of the participants who reported that they use solid fuel for cooking (53.5%) lived in rural areas (71 vs. 37.9%), were illiterate (63.1 vs. 51.6%), had low annual income (45.6 vs. 19.3%), currently smoking (20.3 vs. 17.8%), never drink (69.6 vs. 65.0%), had higher dietary diversity scores (32.5 vs. 18.1%), were underweight (26.7 vs. 21.6%), seldom exercised (28.5 vs. 35.2%), had no IADL and ADL disabilities, had cognitive impairment (30.7 vs. 24.0%), and had lower proportion of chronic diseases (33.08 vs. 42.59%) compared with those who used clean fuel.

**Table 1:** Characteristics of the study participants according to cooking fuel used at baseline.

**Cooking fuels**	**Clean fuels**	**Solid fuels**	**Total**	***P***
	***N*** **= 2,667**	***N*** **= 3,065**	***N*** **= 5,732**	
**Age**	84.5 (10.8)	85.0 (11.3)	84.8 (11.1)	0.13
**Sex**				0.89
Male	1,234 (46.3%)	1,424 (46.5%)	2,658 (46.4%)	
Female	1,433 (53.7%)	1,641 (53.5%)	3,074 (53.6%)	
**Residence**				<0.001
Rural	1,010 (37.9%)	2,177 (71.0%)	3,187 (55.6%)	
City	1,657 (62.1%)	888 (29.0%)	2,545 (44.4%)	
**Education level**				<0.001
Illiterate (0 year)	1,374 (51.6%)	1,929 (63.1%)	3,303 (57.7%)	
Primary school (1–6 years)	900 (33.8%)	917 (30.0%)	1,817 (31.8%)	
High school or higher (>6 years)	388 (14.6%)	213 (7.0%)	601 (10.5%)	
**Current marital status**				0.29
Never married	1,641 (61.8%)	1,846 (60.4%)	3,487 (61.0%)	
Married	1,016 (38.2%)	1,211 (39.6%)	2,227 (39.0%)	
**Family annual income (Yuan)**				<0.001
<30,000	506 (19.3%)	1,371 (45.6%)	1,877 (33.4%)	
30,000–50,000	958 (36.6%)	1,112 (37.0%)	2,070 (36.8%)	
>50,000	1,151 (44.0%)	523 (17.4%)	1,674 (29.8%)	
**Smoking status**				<0.001
Never	1,715 (64.6%)	2,000 (65.6%)	3,715 (65.1%)	
Former	468 (17.6%)	432 (14.2%)	900 (15.8%)	
Current	473 (17.8%)	618 (20.3%)	1,091 (19.1%)	
**Drinking status**				<0.001
Never	1,719 (65.0%)	2,118 (69.6%)	3,837 (67.5%)	
Former	434 (16.4%)	376 (12.4%)	810 (14.2%)	
Current	491 (18.6%)	550 (18.1%)	1,041 (18.3%)	
**Dietary diversity scores^b^**				<0.001
Low	1,369 (51.3%)	957 (31.2%)	2,327 (40.6%)	
Medium	816 (30.6%)	1,112 (36.3%)	1,928 (33.6%)	
High	482 (18.1%)	996 (32.5%)	1,478 (25.8%)	
**BMI**				<0.001
Underweight	543 (21.6%)	782 (26.7%)	1,325 (24.3%)	
Normal	1,323 (52.6%)	1,630 (55.7%)	2,953 (54.2%)	
Overweight/Obese	651 (25.9%)	516 (17.6%)	1,167 (21.4%)	
**Social activity^c^**				<0.001
Low	1,169 (43.8%)	1,534 (50.0%)	2,704 (47.2%)	
Medium	560 (21.0%)	656 (21.4%)	1,216 (21.2%)	
High	938 (35.2%)	875 (28.5%)	1,813 (31.6%)	
**Population-weighted PM**_**2.5**_ **[ug/m**^**3**^**]**				<0.001
≤ 50	1,059 (39.7%)	925 (30.2%)	1,985 (34.6%)	
>50	1,608 (60.3%)	2,140 (69.8%)	3,748 (65.4%)	
**IADL^d^**				0.65
Normal/partial dependence	1,527 (57.3%)	1,773 (57.8%)	3,301 (57.6%)	
Complete dependence	1,140 (42.7%)	1,292 (42.2%)	2,432 (42.4%)	
**ADL^d^**				0.002
Normal/partial dependence	2,253 (84.5%)	2,677 (87.3%)	4,931 (86.0%)	
Complete dependence	414 (15.5%)	388 (12.7%)	802 (14.0%)	
**City population**				0.009
≤ 8 million	2,105 (81.3%)	2,304 (78.4%)	4,409 (79.8%)	
>8 million	485 (18.7%)	633 (21.6%)	1,118 (20.2%)	
**Cognitive function^a^**				<0.001
Normal	2,026 (76.0%)	2,125 (69.3%)	4,152 (72.4%)	
Cognitive impairment	641 (24.0%)	940 (30.7%)	1,581 (27.6%)	
**Chronic disease^e^**				<0.001
No	1,531 (57.41%)	2,051 (66.92%)	3,583 (62.5%)	
Yes	1,136 (42.59%)	1,014 (33.08%)	2,150 (37.5%)	

a *Cognitive impairment was validated according to MMSE scores and education level: Illiterate (0 year) and MMSE <18; Primary school (1–6 years) and MMSE <21; High school or higher (>6 years) and MMSE <25*.

b *Dietary diversity scores were divided by tertiles*.

c *Social activity score was calculated by eight kind of activities (taking in homework, growing vegetable, cleaning the garden, reading newspaper, raising pets, participating in Mahjong, listening to video and interacting with others) and each item was score at 1 “never,” 2 “sometimes,” 3 “always.” The scores range from 8 to 24 with the higher score indicated more social function, and the total score was divided into three-class by tertiles*.

d *Complete dependence of IADL and ADL was defined as last one item need always others to provide assistant*.

e *Four kinds of chronic diseases were included (hypertension, diabetes, stroke and heart disease)*.

To further explore the characteristics of subjects, we presented the mortality and mortality risk ratios using different fuels by gender (Supplementary Table 1) and smoking status (Supplementary Table 2). During the follow-up period from 2011 to 2018, the mortality rate for women was 56.49 (per 1,000), while that for men was 52.36 (per 1,000). Supplementary Table 2 shows that participants using solid fuel who were never smokers (OR = 1.13, CI = 1.03–1.24) or former smokers (OR = 1.21, CI = 0.99–1.47) had higher risk of mortality than those who were smokers (OR = 0.97, CI = 0.82–1.15) 13 and 21%, respectively.

### Association Between Type of Fuel Used for Cooking and Mortality

In the 2011–2018 cohort, a total of 3,240 death cases were ascertained during the 8-year follow-up period. Among these cases, 1,497 and 1,743 used clean fuel and solid fuel, and the mortality was 9.35 and 9.47 per 100 person-years, respectively. The full Cox regression model showed that participants who used solid fuel for cooking had a 9% higher risk of mortality than those who used clean fuel (HR = 1.09, 95% CI = 1.01–1.18) (Table 2). In the 2014–2018 cohort, a total of 1,336 deaths were ascertained: 488, 509, and 339 deaths were recorded for those who used stable clean fuel, stable solid fuel, and switching solid fuel to clean fuel for cooking, respectively. The mortality in these groups was 4.71, 5.07, and 5.42 per 100 person-years, respectively. The full Cox regression model showed that 339 reported a switch from solid to clean fuels and they were not at increased mortality risk relative to the 488 people that reported a stable use of clean fuels (HR = 1.14, 95% CI = 0.99–1.31) although the estimated HR was similar to the one for stable solid fuel users (HR = 1.19, 95%CI = 1.04 = 1.36 *n* = 509) (Table 3).

**Table 2:** Association of cooking fuels with mortality.

**Model**	**Fuel**	
	**Clean fuels**	**Solid fuels**
**Cox regression, HR for mortality (95%CI)**		
Crude, unadjusted model	Ref.	**1.17 (1.10–1.25)**
Model 1	Ref.	**1.15 (1.08–1.23)**
Model 2	Ref.	**1.10 (1.02–1.18)**
Model 3	Ref.	**1.08 (1.009–1.17)**
Model 4	Ref.	**1.09 (1.01–1.18)**
N event	1,497	1,743
Mortality (per 100 person-year)	9.35	9.47

*Adjustments: Model 1: Sex, age; Model 2: further adjusted residence, smoking, alcohol drinking, dietary diversity score, BMI, marital status, social activity and education; Model 3: further adjusted income, PM 2.5 and city population; Model 4: further adjusted IADL, ADL, MMSE, and chronic disease*.

*Bold represents p < 0.05*.

**Table 3:** Association of the change of cooking fuels during 2011–2014 with 2018 mortality.

**Model**	**Fuel**		
	**Stable clean fuels**	**Stable Solid fuels**	**Switching solid fuels to clean fuels**
**Cox regression, HR for mortality (95%CI)**			
Crude, unadjusted model	Ref.	**1.37 (1.23, 1.53)**	**1.38 (1.22, 1.57)**
Model 1	Ref.	**1.33 (1.19, 1.49)**	**1.22 (1.07, 1.39)**
Model 2	Ref.	**1.22 (1.08, 1.38)**	1.10 (0.96, 1.26)
Model 3	Ref.	**1.20 (1.06, 1.37)**	1.11 (0.96, 1.28)
Model 4	Ref.	**1.19 (1.04, 1.36)**	1.14 (0.99, 1.31)
N event	488	509	339
Mortality (per 100 person-year)	4.71	5.07	5.42

*Stable use: persistent use with clean or solid fuels during 2011–2014*.

*Adjustments: Model 1: Sex, age; Model 2: further adjusted residence, smoking, alcohol drinking, dietary diversity score, BMI, marital status, social activity and education; Model 3: further adjusted income, PM 2.5 and city population; Model 4: further adjusted IADL, ADL, MMSE and chronic disease*.

*Bold represents p < 0.05*.

### Subgroup Analysis

**Figure 2:**
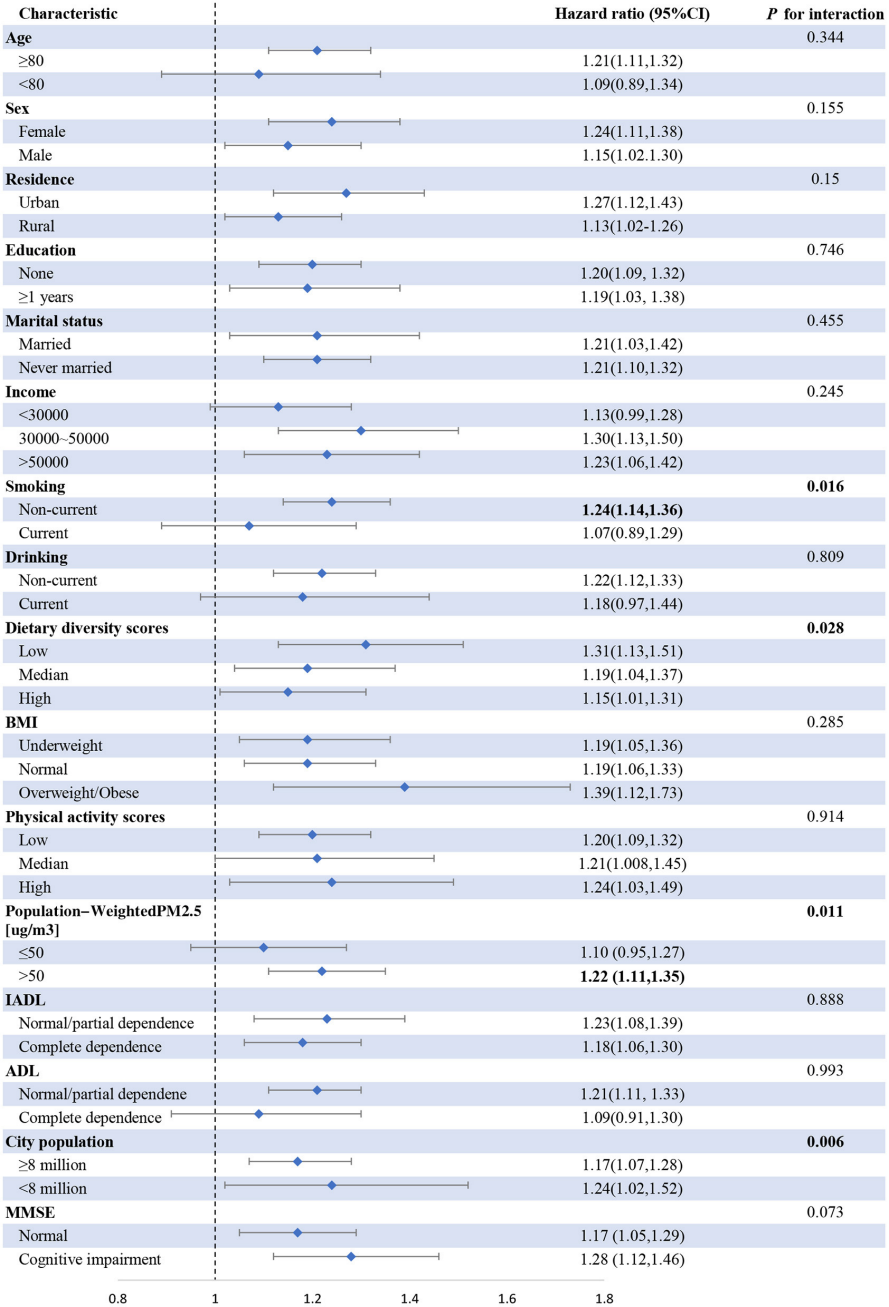
Association of cooking fuel use with mortality among subgroups. Adjustment: sex, age, residence, alcohol drinking, dietary diversity score, BMI, marital status, social activity, education, income and city population, IADL, ADL, MMSE, and chronic disease.

The interaction of 16 modified factors, namely, age, sex, residency, education level, marital status, income level, smoking behavior (Non-current/current), drinking behavior, dietary diversity scores, BMI, physical activity scores, PM_2.5_ concentration, city population, IADL, ADL, and MMSE, was analyzed (Figure 2). The use of solid fuel for cooking was found to be positively associated with mortality across subgroups. The interaction between the use of solid fuel for cooking with smoking behavior (Non-current: 1.24 [1.14, 1.36] vs. current smoker: 1.07 [0.89, 1.29]; *P* for interaction = 0.016), dietary diversity scores (low: 1.31 [1.13, 1.51] vs. median: 1.19 [1.04, 1.37] vs. high: 1.15 [1.01, 1.31]; *P* for interaction = 0.028), PM_2.5_ concentration (≤50 μg/m^3^: 1.10 [0.95, 1.27] vs. >50 μg/m^3^: 1.22 [1.11, 1.35]; *P* for interaction = 0.011) was significant. In general, the relationship was more pronounced in individuals who were currently not smoking (former smokers and ex-smokers), had low dietary diversity scores, who lived in areas with high PM_2.5_ concentrations (>50 μg/m^3^), and living in cities with a low population size (<8 million).

**Figure 3:**
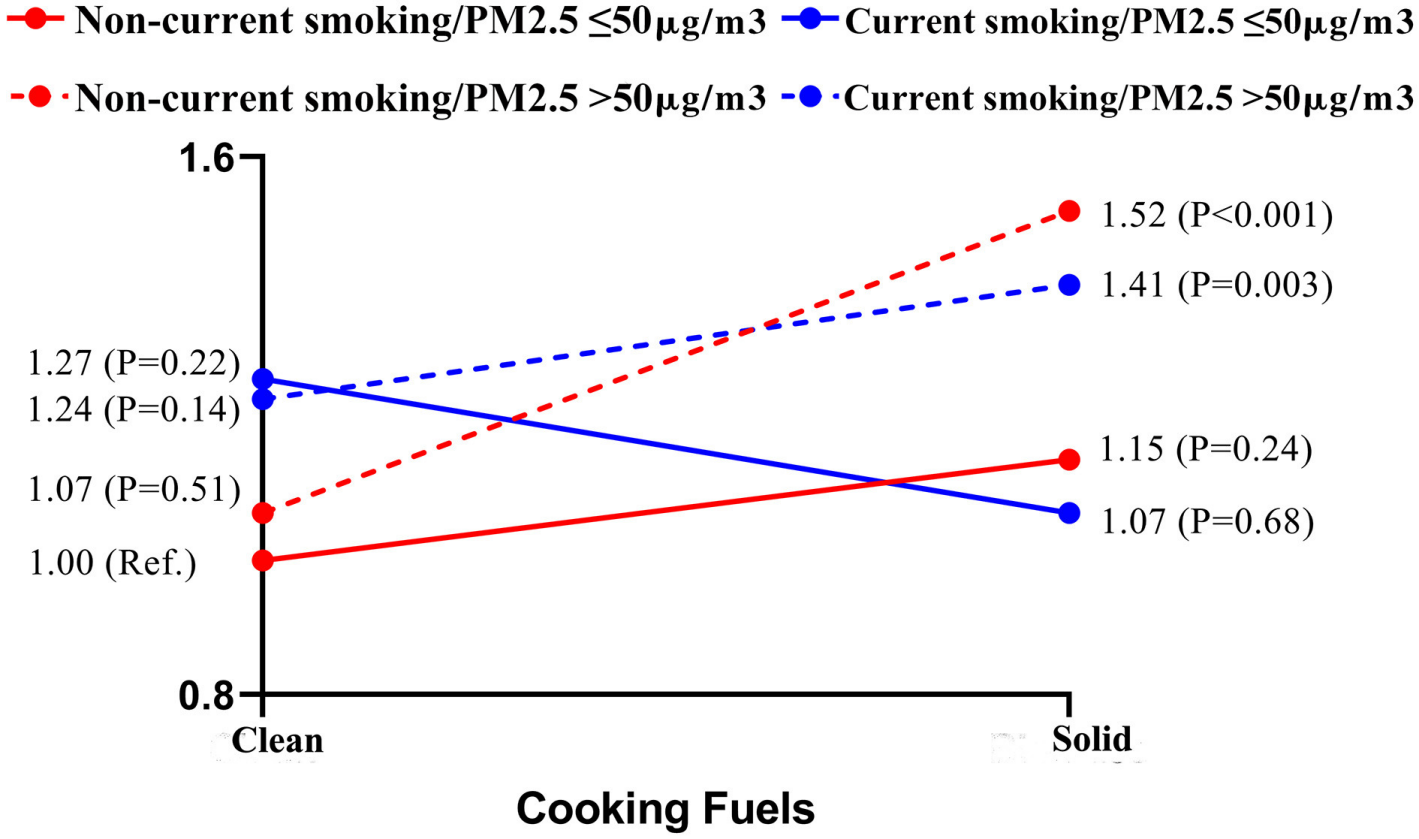
Three-way interaction of cooking fuel use, smoking, concentration of PM_2.5_ on mortality. Adjustment: Sex, age, residence, alcohol drinking, dietary diversity score, BMI, marital status, social activity, education, income and city population, IADL, ADL, MMSE, and chronic disease.

The mechanism of this interaction was further explored. A three-way interaction analysis was performed to directly show the modification effects of PM_2.5_ concentration and smoking status on the type of fuel used for cooking and mortality. The results supported the available evidence that people who used solid fuel, lived in areas with ambient PM_2.5_ concentration of >50 μg/m^3^, and did not smoke (*P* < 0.001) and currently smoked (*P* = 0.003) had higher HRs than those who used clean fuel for cooking, did not smoke, and lived in areas with ambient PM_2.5_ concentration of ≤50 μg/m^3^ (Ref.) (Figure 3).

### Sensitivity Analysis

In sensitivity analyses, the increased mortality risk for solid fuel vs. clean fuel was still observed when individuals lost to follow-up were censored at the last wave (HR = 1.17, 95% CI = 1.08, 1.27) and when individuals who died prior to the second wave (2014) were excluded (HR = 1.28, 95% CI = 1.13, 1.45). Finally, after imputing missing values, those who used solid fuel for cooking had 11% higher hazard risk of mortality than those who used clean fuel (HR = 1.11, 95% CI = 1.02, 1.19) (Table 4).

**Table 4:** Sensitivity analyses for the association of cooking fuels with 8-year mortality.

	**Hazard ratio (95%CI)**		
**Cooking fuel**	Model 1^*^	**Model 2^a^**	**Model 3^b^**
Clean fuel	Ref.	Ref.	Ref.
Solid fuel	1.17 (1.08, 1.27)	1.28 (1.13, 1.45)	1.11 (1.02, 1.19)

*In models 1, 2 and 3, we adjusted for baseline sex, age, residence, smoking, alcohol drinking, dietary diversity score, BMI, marital status, social activity and education, income, PM 2.5 and city population, IADL, ADL, MMSE and chronic disease*.

* *Participants lost to follow-up were treated as censored at the end of the study*.

a *Exclusion of deaths that occurred in the first year of follow-up*.

b *Multiple imputation conducted for missing data*.

## Discussion

In this prospective cohort study of 5,732 Chinese adults aged ≥65 years, the association of the type of fuel used in cooking and all-cause mortality was investigated. Moreover, the potential interaction effects of smoking behavior and PM_2.5_ concentration on all-cause mortality was assessed. The results showed that participants who used solid fuel for cooking had higher mortality risk than those who used clean fuel. However, a follow-up survey revealed that the effects of the use of mixed fuel for cooking on all-cause mortality were not significant. An 8-year follow-up period showed that participants who used solid fuel had 9% higher mortality risk than those who used clean fuel. A 5-year follow-up period showed that persistent solid fuel users were not at increased mortality risk relative to the users that reported a stable use of clean fuels (HR = 1.14, 95% CI = 0.99–1.31). In addition, the association was more pronounced in participants who lived in areas with high ambient PM_2.5_ concentrations and were non-smokers, providing the growing body of evidence on the adverse effects of indoor and outdoor pollutants. The present study aimed to investigate and demonstrate the potential association between the type of fuel used in cooking and mortality among the elderly in LMICs.

The present study supported the findings of previous works and established a positive association between the use of solid fuel and mortality (27, 28). A large-scale cohort of 277,838 Chinese who never smoked and had no prior major disease at baseline indicated that the use of solid fuel is associated with high mortality due to respiratory diseases. People aged ≥60 years had a higher hazard risk for major respiratory diseases (HR = 1.53) than younger people (HR = 1.23 and HR = 1.37) (27). The CKB study also found that the use of solid fuel for cooking and heating increases the mortality due to COPD (28). Furthermore, self-reported information on household cooking and heating was collected at baseline and followed up for 7–11 years. The results showed that coal and wood, the main sources of energy for cooking, pose high risks to the pathogenesis of COPD. However, the Prospective Urban Rural Epidemiology (PURE) study, which recruited 91,350 people across 11 countries, did not support the assertion that the use of solid fuel for cooking in urban regions increases the risk of all-cause, cardiovascular, and respiratory mortality. By contrast, the PURE study obtained the opposite results in rural regions (18). Therefore, in the present study, the effects of the interaction between residence (urban vs. rural areas), the type of fuel used for cooking, and mortality were further assessed to reconcile the conflicting results of the aforementioned studies. The present results did not support the assertion that regional differences are a potential moderator (*P* for interaction = 0.15). The CKB study, which had a larger sample size in urban areas (171,677) than the PURE study (43,001), reported that the use of solid fuel for cooking is significantly associated with high risk for all-cause and cardiopulmonary mortality (12, 18). Recently, the “urban-rural return migration” has become popular, which conforms to the old saying in traditional Chinese culture that “falling leaves settle on the roots” (29, 30). According to previous studies, more and more elderly people living in cities are more willing to move to rural areas with age. In general, the environment in rural areas is better than that in urban areas in terms of pollution level. Thus, rural areas are an ideal environment for the elderly. Increased health awareness may induce the elderly to utilize clean energy rather than traditional energy. In this background, the difference between urban and rural areas may not be remarkable. The present findings may be used as an invaluable reference for guiding the efforts of the government in subdividing areas and for formulating targeted policy to control all-cause mortality due to pollution.

Among participants in the 2014–2018 survey, 339 reported a switch from solid to clean fuels and they were not at increased mortality risk relative to the 488 people that reported a stable use of clean fuels (HR = 1.14, 95% CI = 0.99–1.31) although the estimated HR was similar to the one for stable solid fuel users (HR = 1.19, 95%CI = 1.04 = 1.36 *n* = 509). Most studies focused on the long-term effects of the use of solid fuel on mortality. However, as housing conditions improve and health awareness increases, the type of fuel used for cooking changes over time. The scientific community must determine the mechanism of this effect within a social context. A prospective cohort that covered five urban areas and involved 226,186 participants that were followed up for 10 years reported that cessation of the use of solid fuel for cooking decreases the excess risk of all-cause and cardiopulmonary mortality by over 60% (12). Another study that focused on rural areas in China found that individuals who switched from solid fuel to clean fuel have a lower risk of cardiovascular and all-cause mortality (4). The present results show that cessation of solid fuel usage offsets the long-term detrimental effects of this practice among the elderly. Although many Chinese households continue to use traditional energy sources, such as coal- and wood-burning stoves, China is ahead of most LMICs in terms of energy transition. Along with rapid urbanization, hundreds of millions of rural homes have started utilizing clean fuel, such as natural gas and electricity (31). In a cohort of over 7000 homes in three provinces (Beijing, Shanxi, and Guangxi), researchers used photograph-based questionnaires to identify the type of stove used at home, determined the frequency and timing of its use, and classified the type of fuel employed in cooking (32). They found that participants who stopped using solid fuel were younger and more educated, but had poor self-reported health. Moreover, they found that individuals were more likely to adopt clean technology if they were younger or retired, lived in smaller households, and had a higher income (32). With the goal of achieving health and wellness across the entire country, living standards and residential settings will generally and considerably improve. China will increase its share of consumption of non-fossil fuel to about 20% of primary energy sources by 2030; by then, non-fossil fuel and natural gas will become the main energy sources of the country (Resolve to shift to clean fuel must not waver 2017). The present study also found that participants who stopped using solid fuel for cooking were in pre-mortality status or had comorbidities. This situation might have influenced the present results; thus, the HR of switching from solid fuel to clean fuel was not significant (12). The mechanism by which switching from solid fuel to clean fuel compensates for the reduction in health hazards must be explored.

Several biological mechanisms may explain why and how solid fuel adversely affect the elderly. Indoor combustion of solid fuel is always associated with poor ventilation and inefficient release of poisonous and harmful substances (33). The concentration of indoor particulate matter substantially increases when solid fuel is used for cooking; as a consequence, residents become exposed to heavy metals, PAHs, carbon monoxide, nitric oxide, and formaldehyde (34). Various physiological processes, such as the production of reactive oxygen species (ROS), oxidative stress, and systemic inflammation, are some of the adverse effects of the combustion of solid fuel (35). For example, PAHs are carcinogens that originate from the incomplete combustion of organic matter, whose essential metabolites are phenolic compounds with hydroxy groups (36). The electrophilic metabolites of PAHs are prone to combine and interact with ROS, thereby disrupting human proteins, esters, and DNA and causing oxidative damage (36). These environmental factors may accelerate cardiomyocyte apoptosis and increase the risk of adverse cardiovascular events by promoting the release of inflammatory factors and chemokines through oxidative damage (37–39). Chronic exposure to the smoke from the combustion of solid fuel may produce leukocyte-platelet aggregates, which may potentially lead to unstable angina and apoplexy (40). Moreover, such exposure may lead to dysfunctional diastole and heart contraction (41). Women who are exposed to the smoke coming from burning wood suffer from high systolic blood pressure and non-corresponding ST-segment depression (42). Additionally, solid fuels have indirect effects on mortality by affecting neuropsychological and cognitive functions. A study conducted in China reported that individuals exposed to burning solid fuel had low visuoconstruction scores and episodic memory dimensions of cognitive ability (43). The pollutants of solid fuel can activate inflammatory reactions in the human body. Inflammatory factors that enter the human brain through the blood-brain barrier damage brain functions (44). Particles break the tight junction of capillary endothelial cells in brain tissues, causing the aggregation of β-amyloid plaques and α-synuclein and releasing inflammatory factors, which ultimately impair the nervous system (45). Toxic materials can interact with microglia, astrocytes, and neurons in brain tissues to induce inflammatory response or oxidative stress, leading to neurotoxicity (46). A previous study indicated that rapid cognitive decline is linked to high mortality, and participants aged between 65 and 79 years with normal cognitive scores were enrolled (47). The present study found that the association between the type of fuel used for cooking and mortality was not significant among participants who currently do not smoke. A plausible explanation for this result was that non-smoking elderly were more sensitive to the effects of pollutants coming from burning solid fuel, and these effects were attenuated among those who smoked because of long-term exposure to smoke. However, the reasons why non-smokers are more vulnerable to solid fuel damage are unclear and require further research. This finding highlighted the essential role of health interventions to prevent smoking in the general population.

The present study also found that the association between the use of solid fuel for cooking and mortality was the strongest among those who did not smoke and were exposed to ambient PM_2.5_ concentration of >50 μg/m^3^. The effects of air pollution on the human body are a result of the interaction between indoor and outdoor pollutants. A study explored whether indoor air pollution can modify the effects of TRAP. It found that the elderly who lived closer to major roadways had an increased risk for cognitive impairment, and solid fuel synergistically enhanced the TRAP effect (15). The present study showed that the association between the use of solid fuel for cooking and mortality was more pronounced in environments with high PM_2.5_ concentrations regardless of smoking behavior. The effects of solid fuel may be lumped with non-stratified samples, and industrialized cities and economies based on fossil fuel energy should focus on environmental epidemiology. The present results also indicated that people who do not smoke had a higher hazard risk for mortality than those who smoke when the former were simultaneously exposed to burning solid fuel and high PM_2.5_ concentrations. This result suggested that smoking and PM_2.5_ exposure synergistically affected the association between the use of solid fuel and mortality because smoking adversely affects the nervous, cardiovascular, and respiratory systems (48, 49). However, the present study suggested that the effects of smoking may be weakened by PM_2.5_ concentration and the type of fuel used for cooking. Another reason is that the CLHLS subjects were probably not representative of the general population but rather of vulnerable elderly people. Therefore, participants suffering from various chronic diseases or pre-mortality should cease smoking and adopt a healthy lifestyle.

Apart from the large sample size and relatively new dataset, the main advantage of this study includes the use of a three-way interactive analysis as a follow-up study to obtain multi-factor synergies between cooking fuel and all-cause mortality, such as PM_2.5_ and smoking status. Moreover, the study supported the association between solid fuel for cooking and all-cause mortality and the alleviative effect of switching from solid fuel use to clean fuel use on all-cause mortality.

The present study has several limitations. First, it did not have sufficient and valid information on cause-specific mortality. Therefore, the mechanism of hypothesis could not be confirmed at the moment. Second, the proxy variable of ventilation was not included in the baseline questionnaire, and the effects of ventilation on the smoke coming from burning fuel for cooking were not explored. Third, owing to the limitation of this study's design, the concentration of pollutants and the type of cooking oil were not considered. Finally, the reality is more complex because the type of fuel used for cooking changed. The CLHLS launched survey waves every 2–3 years, and the switching of cooking fuel might have overlapped.

## Conclusion

The present study established a clear association between the type of solid fuel used for cooking and mortality. The association between mortality risk and solid fuel use was stronger for individuals exposed to high levels of PM_2.5_. These findings add to the growing body of evidence on the adverse effects of indoor and outdoor pollutants. On the basis of these results, this study emphasizes that the elderly must be protected from the risk of mortality and other chronic diseases due to the combustion of solid fuel. Health policies must ensure sustainable and environmentally friendly societies to cater to the aging population of China.

## Data Availability Statement

The CLHLS questionnaires are available at https://sites.duke.edu/centerforaging/programs/chinese-longitudinal-healthy-longevity-survey-clhls/survey-documentation/questionnaires/. The full datasets used in this analysis are available from the corresponding author upon reasonable request.

## Ethics Statement

The datasets that support this article are publicly available from the project of the CLHLS. It was approved by research Ethics Committees of Peking University (IRB00001052-13074). The datasets analyzed during the current study are available online (http://opendata.pku.edu.cn/) from Peking University Open Research Data for researchers who meet the criteria for access to these de-identified data. No experimental interventions were performed. Written informed consent was obtained from the individual(s) for the publication of any potentially identifiable images or data included in this article.

## Author Contributions

SS: conceptualization, writing—original draft, and formal analysis. ML: conceptualization, writing—original draft and visualization, and formal analysis. XM: validation and writing—review and editing. YD and SC: conceptualization and writing—review and editing. All authors contributed to the article and approved the submitted version.

## Funding

Data used in this research were provided by the study entitled Chinese Longitudinal Longevity Survey (CLHLS) was jointly implemented by the Center for Healthy Aging and Development Studies of Peking University and Duke University. CLHLS was supported by funds from the U.S. National Institutes on Aging (NIA), China Natural Science Foundation, China Social Science Foundation, and UNFPA.

## Conflict of Interest

The authors declare that the research was conducted in the absence of any commercial or financial relationships that could be construed as a potential conflict of interest.

## Publisher's Note

All claims expressed in this article are solely those of the authors and do not necessarily represent those of their affiliated organizations, or those of the publisher, the editors and the reviewers. Any product that may be evaluated in this article, or claim that may be made by its manufacturer, is not guaranteed or endorsed by the publisher.

## Footnotes

^1^Chinese EPA. *Chinese Environmental Protection Agency* (2019). Available online at: https://air.cnemc.cn:18007/ (accessed November 10, 2019).
