# Supplementary material for: All-Cause Mortality Risk Associated With Solid Fuel Use Among Chinese Elderly People: A National Retrospective Longitudinal Study
Source: Front Public Health. 2021 Oct 14;9:741637. doi: 10.3389/fpubh.2021.741637 (PMC8551618; doi:10.3389/fpubh.2021.741637)
Supplement: Supplementary file 1 [file Table_1.DOCX]

| Table S1. Association of cooking fuels with sex |  |  |
| --- | --- | --- |
|  | **Sex** | |
|  | **male** | **female** |
|  |  |  |
| **Mortality(per 1000)** | **52.36(49.75-55.12)** | **56.49(53.93-59.18)** |
|  |  |  |

| Table S2. Association of cooking fuels with smoking status | | |
| --- | --- | --- |
| **Smoking status** | | **OR(95%CI)** |
|  |  |  |
| **Never** |  |  |
|  | **Clean fuels** | Ref. |
|  | **Solid fuels** | 1.13(1.03-1.24)** |
| **Former** |  |  |
|  | **Clean fuels** | Ref. |
|  | **Solid fuels** | 1.21(0.99-1.47)* |
| **Current** |  |  |
|  | **Clean fuels** | Ref. |
|  | **Solid fuels** | 0.97(0.82-1.15) |
|  |  |  |

*** p<.01, ** p<.05, * p<.1
