# Supplementary material for: All-Cause Mortality Risk Associated With Solid Fuel Use Among Chinese Elderly People: A National Retrospective Longitudinal Study
Source: Front Public Health. 2021 Oct 14;9:741637. doi: 10.3389/fpubh.2021.741637 (PMC8551618; doi:10.3389/fpubh.2021.741637)
Supplement: Supplementary file 2 [file Data_Sheet_1.DOCX]

**Appendix Methods.**

**Detailed definitions of covariates in our study.**

Age (years) was calculated based on difference between the follow-up year and Georgian calendar dates. Current residence was dichotomized as “Rural area” and “City area”. Education level was divided based on years of schooling (0 year, 1-6 year and >6 years). Current marital status was grouped into “Never married” (widowed, separated, divorced, or never married) and “Married”. Annual family income was classified by tertiles (<30,000, 30,000-50,000, >50,000). Smoking and drinking status was defined as “Never”, “Formal” and “Current”. Dietary diversity scores were calculated according to eight food group (staple food, fresh fruit and vegetable, meat, fish, sugar, milk and nuts). Types of staple food included rice, corn, wheat, half rice and half wheat. Amount of staple food was grouped by quintile and meant scores from 1 to 5. In addition, a score of 0 indicated that individual did not took grain as staple food. The frequency of fruit and vegetable intake was coded as “every day or almost every”, “quite often”, “occasionally” and “rarely or never”. The scores ranged from 1 to 4, with higher points reflecting higher times for intake. The rest five food were recorded as “almost every day”, “at least once per week”, “at least once per month”, “occasionally” and “never”. The scores for these food groups ranged from 1 to 5 with higher scores representing higher frequency for intake. Therefore, a total of dietary diversity score was summed for all eight foods with ranging from 7 to 38 with higher scores equaling to abundant dietary diversity (Wang et al. 2020). Body mass index (BMI) was calculated as weight in kilograms divided by height without shoes in meters squared. Social activity Social activity score was calculated by eight kind of activities (taking in homework, growing vegetable, cleaning the garden, reading newspaper, raising pets, participating in Mahjong, listening to video and interacting with others) and each item was score at 1 “never”, 2 “sometimes”, 3 “always”. The scores range from 8 to 24 with the higher score indicated more social function, and the total score was divided into three-class by tertiles. City population was dichotomized into “>8 million” vs. “≤8 million”. ADL were measured with the following six items: (1) Bathing; (2) Dressing; (3) Toileting; (4) Indoor moving; (5) Continence of defecation; (6) Eating. Each item was scored from 1 to 3 (1 score representing complete independence; 2 scores representing partially dependence; 3 scores representing complete dependence). The more scores the respondents obtained, the higher ADL dependence they would be. IADL were rated with eight questions: (1) Can you visit your neighbors by yourself? (2) Can you go shopping by yourself ? (3) Can you cook a meal by yourself when necessary? (4) Can you wash clothes by yourself when necessary? (5) Can you walk a kilometer at a time by yourself? (6) Can you lift a weight of 5 kg, such as a heavy bag of groceries? (7) Can you continuously squat and stand up three times? (8) Can you take public transportation by yourself? Item were rated on a three-point scale ranging from 1(Complete independence) to 3 (Complete dependence). In current study, participants were regarded as complete dependence when the score of IADL or ADL was 3 point (Zhang et al. 2021). Chinese version of the Mini Mental State Examination (MMSE) was used to evaluate the global cognitive function. MMSE has four dimensions of cognitive orientation, calculation, recall and language capacity, with a total of 24 items scoring from 0 to 30, and the higher scores indicate a higher level of dependence for the respondents (Zeng et al. 2010). The elderly who obtained 24 scores and above were defined as “normal cognitive function”, while those scored less than 24 were evaluated to be “cognitive impairment” (Lei et al. 2020). Four common chronic diseases were considered as adjusted variables: hypertension, diabetes, heart disease and stroke. Based on residence of participants, the city-level concentration of ambient PM _2.5_ was estimated from Atmospheric Composition Analysis Group (ACAG. 2020). Previous researches have illustrated the detailed process and algorithm (Kalashnikova et al. 2020; van Donkelaar et al. 2019). The surface PM _2.5_ concentration was estimated by combining satellite-retrieved aerosol optical depth (AOD) with four satellite instruments (twin MODIS instruments, the MISR instrument and the SeaWiFS instrument). According to previous paper, annual mean geophysical PM _2.5_ estimates were highly consistent with globally distributed ground monitors (R2=0.81; slope=0.90) (Kalashnikova et al. 2020). Geographically weighted regression indicated that higher cross validated agreement was linked with ground monitors (R2 = 0.90-0.92; slope = 0.90-0.97) ( Kalashnikova et al. 2020).

**Reference**

Wang Z, Pang Y, Liu J, Wang J, Xie Z, Huang T. Association of healthy lifestyle with cognitive function among Chinese older adults. Eur J Clin Nutr. 2020 Oct 28.

Zhang Y, Xiong Y, Yu Q, Shen S, Chen L, Lei X. The activity of daily living (ADL) subgroups and health impairment among Chinese elderly: a latent profile analysis. BMC Geriatr. 2021 Jan 7;21(1):30.

Yi Z, Vaupel JW. Functional capacity and self–evaluation of health and life of oldest old in China. J Soc Issues. 2010;58(4):733–48.

Lei X, Bai C. Cognitive function and mental health of elderly people in China: findings from 2018 CLHLS survey. China Popul Dev Stud. 2020;3:343–51.

Atmospheric Composition Analysis Group. Surface PM _2.5._

Website: http://fizz.phys.dal.ca/~atmos/martin/?page_id=140

Hammer MS, van Donkelaar A, Li C, Lyapustin A, Sayer AM, Hsu NC, Levy RC, Garay MJ, Kalashnikova OV, Kahn RA, Brauer M, Apte JS, Henze DK, Zhang L, Zhang Q, Ford B, Pierce JR, Martin RV. Global Estimates and Long-Term Trends of Fine Particulate Matter Concentrations (1998-2018). Environ Sci Technol. 2020 Jul 7;54(13):7879-7890.

van Donkelaar A, Martin RV, Li C, Burnett RT. Regional Estimates of Chemical Composition of Fine Particulate Matter Using a Combined Geoscience-Statistical Method with Information from Satellites, Models, and Monitors. Environ Sci Technol. 2019 Mar 5;53(5):2595-2611.
