# Supplementary figures and images for: All-Cause Mortality Risk Associated With Solid Fuel Use Among Chinese Elderly People: A National Retrospective Longitudinal Study
Source: Front Public Health. 2021 Oct 14;9:741637. doi: 10.3389/fpubh.2021.741637 (PMC8551618; doi:10.3389/fpubh.2021.741637)

**Appendix Formula.**


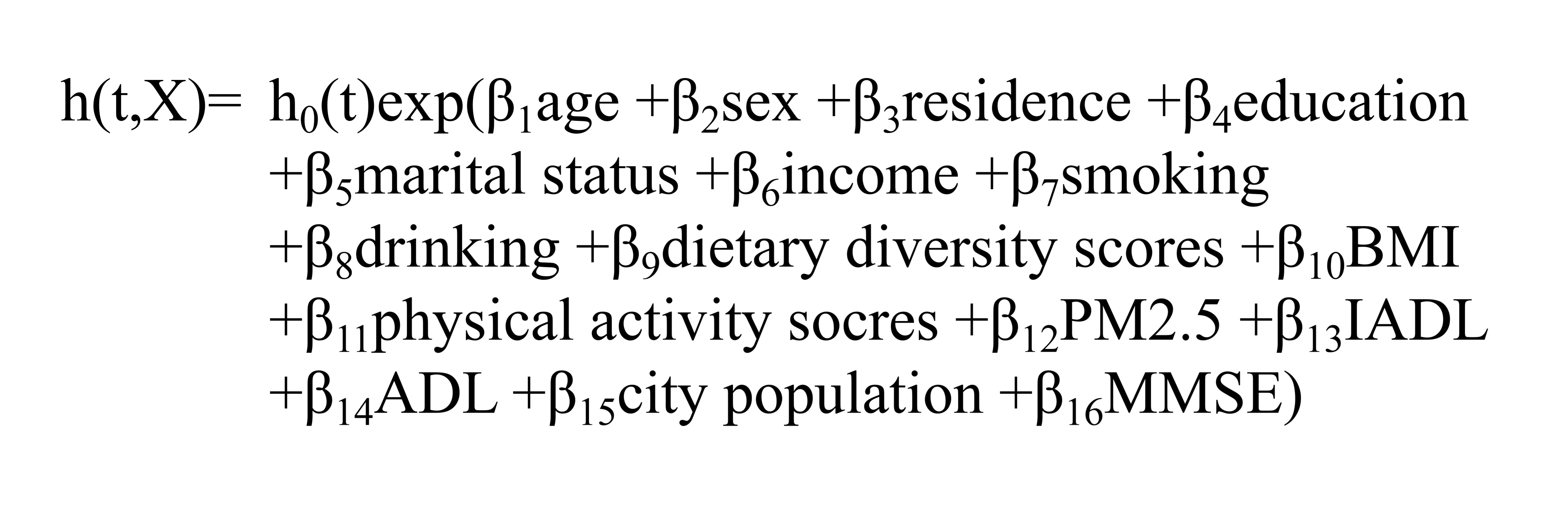

Supplement: Supplementary file 3 [file Data_Sheet_2.DOCX]

Supplementary Figure 1: The timeline of survey stages


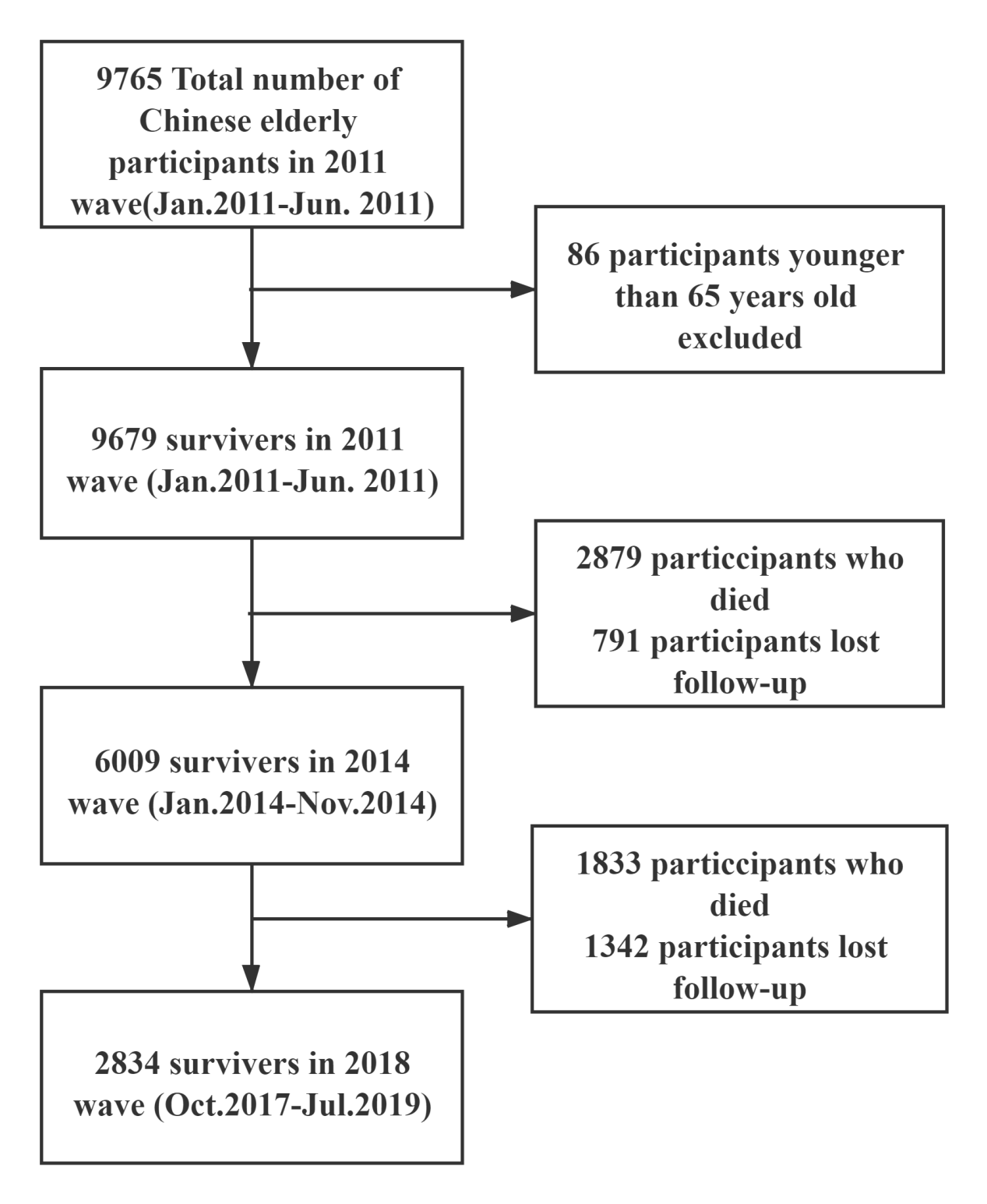

Supplement: Supplementary file 4 [file Data_Sheet_3.DOCX]
